# The Long-term Visual Outcomes of Primary Congenital Glaucoma

**DOI:** 10.18502/jovr.v15i3.7451

**Published:** 2020-07-29

**Authors:** Hamed Esfandiari, Alisa Prager, Kiana Hassanpour, Sudhi P. Kurup, Rebecca Mets-Halgrimson, Hawke Yoon, Janice Lasky Zeid, Marilyn B. Mets, Bahram Rahmani

**Affiliations:** ^1^Division of Ophthalmology, Ann & Robert H. Lurie Children’s Hospital of Chicago, Chicago, USA; ^2^Department of Ophthalmology, Northwestern University Feinberg School of Medicine, Chicago, USA; ^3^Ophthalmic Research Center, Institutue for Ophthalmology and Vision Science, Shahid Beheshti University of Medical Sciences, Tehran, Iran

**Keywords:** Ab Externo Trabeculotomy, Long-term Outcomes, Primary Congenital Glaucoma, Stereopsis, Visual Function

## Abstract

**Purpose:**

To evaluate the long-term visual outcomes of ab externo trabeculotomy for primary congenital glaucoma (PCG) at a single pediatric ophthalmology center.

**Methods:**

In this retrospective single-center case series, data from 63 eyes of 40 patients who underwent ab externo trabeculotomy between September 2006 and June 2018 were included. The data were analyzed for best corrected visual acuity (BCVA), stereopsis, and surgical success. Kaplan–Meier analysis was performed using the surgical success criteria defined as intraocular pressure (IOP) ≤ 21 mmHg and ≥ 20% below baseline without the need for additional glaucoma surgery.

**Results:**

BCVA at the time of diagnosis was 0.37 ± 0.48 logMAR, which changed to 0.51 ± 0.56 logMAR at the final follow-up (*P* = 0.08). Twenty-five percent of patients had BCVA equal to or better than 20/40 at the final visit. The mean refraction at baseline was –4.78 ± 5.87 diopters, which changed to less myopic refraction of –2.90 ± 3.83 diopters at the final visit. Optical correction was prescribed in 66% of eyes at the final visit. The average final stereopsis was 395.33 sec of arc. The linear regression model showed a significant association between the surgery success rate and final BCVA as well as stereoacuity (*P*-values: 0.04 and 0.03, respectively). Intraocular pressure (IOP) decreased significantly from 29.79 ± 7.67 mmHg at baseline to 16.13 ± 3.41 mmHg at the final follow-up (*P* = 0.001).

**Conclusion:**

Patients with PCG can achieve an acceptable visual acuity and stereoacuity, particularly in cases of timely intervention and close follow-up.

##  INTRODUCTION

Primary congenital glaucoma (PCG) is the most common type of childhood glaucoma and a major cause of blindness in children.^[1,2]^ PCG is secondary to angle dysgenesis and is primarily a surgical condition.^[3]^ Surgical options include angle surgeries, trabeculectomy, glaucoma drainage devices, and cyclodestructive procedures in advanced cases.^[4]^ Historically, the effect of angle surgeries on the intraocular pressure (IOP) has been considered the main outcome measure in most studies on PCG.

While surgical outcomes are thoroughly discussed in the literature, not much is known about the visual outcomes of glaucoma surgery in patients with PCG. Studies have shown that unilateral disease, poor vision at the time of diagnosis, multiple surgeries, and ocular comorbidities are associated with poor visual outcomes.^[5]^ Even after a timely intervention, these patients need treatment and monitoring for amblyopia, which necessitate frequent visits or examination under anesthesia. The final visual outcome is strongly related to early diagnosis and management of amblyopia. Frequent anesthesia or sedation, complexity of the disease, and the associated visual impairment could have significant impact on children's psychological behavior and make the assessment of visual function more complicated.^[6]^ Thus, the ultimate goal of childhood glaucoma management is lifelong control of IOP to maintain visual function.^[7,8]^

The purpose of this study was to report the long-term visual outcomes (including Snellen visual acuity and stereopsis) of patients with PCG, treated with ab externo trabeculotomy at a single center.

##  METHODS

This study was approved by the Institutional Review Board of the Ann & Robert H. Lurie Children's Hospital of Chicago. We followed the tenets of the Declaration of Helsinki and regulations of the Health Insurance Portability and Accountability Act.

Patients who underwent ab externo trabeculotomy for PCG at the Lurie Children's pediatric ophthalmology center between September 2006 and June 2018 were identified using current procedural terminology (CPT) codes and included in the study. We collected data such as preoperative IOP, baseline ocular biometric characteristics including axial length (AL), central corneal thickness (CCT), corneal diameter, presence of Haab's striae, number of preoperative glaucoma medications, type of surgery, and intra- and postoperative complications. The severity of glaucoma was assessed using three parameters: IOP, corneal diameter, and corneal clarity. Each parameter was given a score of 1–3, and the total score decided the severity of PCG: mild (1–3), moderate (4–6), or severe (7–9) PCG.^[9]^ At each postoperative visit, the best corrected visual acuity (BCVA), stereopsis, IOP, glaucoma medications, and complications were noted. Surgical success criteria were defined as IOP ≤ 21 mmHg and ≥ 20% below the baseline without the need for additional glaucoma surgery (except ab externo trabeculotomy). The exclusion criteria were as follows: history of intraocular surgery, combined surgical procedures, any forms of anterior segment dysgenesis, and a follow-up of less than six-months.

Stereopsis was quantitatively assessed with the Titmus test (Stereo Optical Inc., Chicago, IL) with fly and graded circles. During the examination, children wore their correction under the Polaroid lenses, and the examiner used his index finger to guide fixation. Patients were asked to grab the wings of the fly and point out to the circle that seemed to “jump” out of the book. In the case of a wrong answer, the immediately preceding target was repeated. The last correct target identified was used as the child's stereopsis measurement. Vision was measured with age-appropriate methods and converted to logMAR for statistical analysis. All patients underwent complete ophthalmologic and orthoptic evaluation. Cycloplegic refraction were performed by instilling two drops of 1% cyclopentolate hydrochloride in each eye at 5-min intervals.

All surgeries were performed under general anesthesia. A fornix-based localized peritomy was created in the temporal or superior quadrant. Triangular or rectangular 3mm limbal base superficial scleral flap was fashioned, followed by a radial incision to expose the Schlemm canal under high magnification. Scleral cut-down was initiated from the blue zone up to the white zone until aqueous was seen oozing out from the cut ends of the canal. The Harms Trabeculotome was then passed into each end of the Schlemm canal to gently cut through the canal in the anterior chamber. The scleral flap and peritomy were sutured with Vicryl sutures.

All statistical analyses were performed using the SPSS software (SPSS Statistics for Windows, Version 25, IM Corp., Armonk, NY, USA). To compare the change in IOP, we used an interaction analysis within a linear mixed model. To evaluate the baseline differences, we used the *T*-test, Chi-Square, and Fisher's exact tests. Kaplan–Meier survival plots were constructed to assess the long-term survival rates; these were compared using the log-rank test. Linear regression analysis was used to evaluate the factors associated with surgical success.

##  RESULTS

Sixty-three eyes of 40 patients were included in this study. The mean age at diagnosis was 6.8 ± 10.6 months and 62.5% of the patients were male. The mean follow-up time was 85.7 ± 32.9 months (Table 1). The preoperative BCVA was 0.37 ± 0.48, which changed to 0.51 ± 0.56 logMAR at the final follow-up (*P* = 0.08). The baseline visual acuity was measured with fixation and following method in 27 eyes, Teller acuity card in 30, LEA symbols in 4, and HOTV in 2. Twenty-five percent of patients had BCVA ≥ 20/40 at the final visit. The mean refraction of the patients was –4.78 ± 5.87 diopter at baseline, which changed to less myopic refraction of –2.90 ± 3.83 diopter at the final visit. Sixty-six percent of eyes were prescribed optical correction at the final visit. The average final stereopsis was 395.33 sec of arc (range, 40–800). The linear regression model showed a significant association between surgical success and final BCVA as well as stereoacuity (*P*-values: 0.04 and 0.03, respectively). The final stereoacuity corresponded to the final BCVA of 0.61 logMAR. The average baseline IOP was 29.7 ± 7.6 mmHg, which decreased to 16.1 ± 3.4 mmHg at the final visit, corresponding to 44% reduction from the baseline. The Kaplan–Meier survival curves indicated a time to failure of 107.7 ± 9.04 months. Among the baseline parameters, only age at diagnosis of less than three months was associated with a higher failure rate. Of the 63 eyes, 21 met the criteria for mild, 29 for moderate, and 13 for severe PCG. While mild PCGs had significantly better visual acuity and stereopsis at the final follow-up (*P* < 0.05), there was no significant difference between the moderate and severe groups. Thirty-five eyes (56%) underwent repeat trabeculotomy to treat a different area of the trabecular meshwork because of inadequately controlled IOP after the first session. Additional glaucoma surgery (glaucoma shunt procedure) was performed in seven patients.

**Table 1:** Baseline characteristics of the patients.


**Clinical characteristics (Range)**	
Gender	Male	25 (62.5%)
Unilateral disease	Number (%)	17 (42.5%)
Age at the time of diagnosis	Mean ± SD (Months)	6.89 ± 10.68 (0 – 63)
Follow-up	Mean ± SD (Months)	85.74 ± 32.95 (3 – 156)
IOP	Mean ± SD (mmHg)	29.79 ± 7.67 (11 – 54)
C/D ratio	Mean ± SD	0.55 ± 0.22 (0.2 – 0.95)
Corneal diameter	Mean ± SD (mm)	12.79 ± 1.26 (9.50 – 15.5)
Central corneal thickness	Mean ± SD (Microns)	585.44 ± 76.57 (470 – 761)
Axial length	Mean ± SD (mm)	22.37 ± 2.50 (17.50 – 28.50)
Refraction	Mean ± SD (Diopters)	–4.78 ± 5.87 (–25.0 – +5.50)
Time of diagnosis by category	≤ 3 Months	18 (31%)
	3 < Age ≤ 6 months	26 (48%)
	> 6 months	14 (24.1%)
Ocular signs	Presence of Haab striae	45 (72.6%)
	Buphthalmos	30 (83.3%)
Ocular symptoms	Tearing	30 (93.8%)
	Photophobia	24 (82.8%)
SD, standard deviation; IOP, intraocular pressure; C/D ratio, cup to disc ratio		

##  DISCUSSION

The main goal of our study was to determine the long-term visual outcome of ab externo trabeculotomy for PCG. In our study, ab externo trabeculotomy resulted in 44% reduction in the IOP with a long-term success rate of 65%. Our success rate is consistent with those in previous reports, which ranged from 45% to 85%.^[8,9]^ The final visual acuity in our study was 0.51 logMAR and 25% of the patients had corrected vision better than 20/40 at the final visit. The visual outcome in our study is comparable to that of other reports with similar demographic data.^[10,11]^ However, we found better final visual function than that reported previously in the AlDarrab study in which 55% of the patients had BCVA < 20/60.^[6]^ Poor visual outcomes in their study could be explained by more severe glaucoma population, high prevalence of CYP1B1 mutation, delayed diagnosis, and inadequate follow-up.^[12]^

Poor vision in children with PCG is multifactorial; high IOP in early life causes structural changes in the eye such as globe enlargement, corneal edema and opacity, tears in the Descemet's membrane (Haab's striae), high refractive error, anisometropia, and glaucomatous optic neuropathy^[13,14,15]^. Corneal edema was present in almost 55% of patients at the time of diagnosis, which resolved after surgery in all cases, but accompanying Haab's striae, with or without corneal edema, persisted in two-third of our patients. Myopic refraction was the most frequent refractive error at baseline, which decreased with IOP reduction. The correlation between myopia and glaucoma can be attributed to the increase in the axial diameter of the eye as a consequence of high IOP.^[14]^ Sixty-six percent of our patients needed optical correction for myopia or myopic astigmatism at the final visit. Astigmatism is usually related to asymmetric expansion of the anterior chamber, corneal scar, and Haab's striae. In most cases, only optical correction was prescribed, but in 7% of the eyes (5 eyes) optical devices were needed. Telescopic systems (angular magnification) were the most common aids used for improving vision. Lower magnifications are beneficial in constricted visual field and low image illumination.^[13]^ No patient needed optical aid for near vision, probably due to the large range of accommodation in children. Optical devices can improve the visual function and promote daily activities; therefore, they should be employed as early as possible.^[15]^

We did not find any association between the age at diagnosis and final visual acuity. This finding is in contrast to other studies that showed a close relationship between age at PCG diagnosis and visual function.^[13]^ However, we found a positive association between IOP control and final visual function. It is likely that timely intervention in our cohort and close follow-up reversed the detrimental effect of high IOP on the function and structure of the eye.

The average final stereopsis in our study was 395.33 sec of arc. The relationship between visual acuity of the amblyopic eye and stereoacuity is complex.^[16]^ In general, worse visual acuity is associated with worse stereoacuity; however, it largely depends on the etiology of amblyopia; strabismic amblyopes have the worst stereoacuity, while anisometropic amblyopes retain some stereopsis.^[17]^ Furthermore, the presence of stereoacuity in patients with PCG can improve the outcomes of amblyopia treatment.^[18,19]^ The results of our study show that while stereoacuity is not normal, it is nevertheless functional in many patients.

Our study was limited by its retrospective nature, variable follow-up duration, and multiple examiners. Additionally, this study was conducted at a single tertiary academic referral center, and the results may not be generalizable to other practice facilities. Baseline visual acuity measurement was not optimal due to age, which made the comparison of pre- and post-surgery measurements less reliable. However, the aim of our study was to present the long-term outcomes of visual function of PCG. Such data for visual acuity are scarce in the literature and there has been no study on stereopsis outcome in PCG.

In summary, the result of our study showed that patients with PCG can achieve an acceptable visual acuity and stereoacuity with timely intervention and close follow-up. Although our study was not designed to compare the visual function changes after ab externo trabeculotomy, we did not observe any improvement in visual acuity as this parameter could be reliably measured after the procedure. This lack of significant improvement should be discussed preoperatively to gauge the expectations.

##  Financial Support and Sponsorship

None.

##  Conflicts of Interest

There are no conflicts of interest.
